# Heparin-Based Hydrogel Micropatches with Human Adipose-Derived Stem Cells: A Promising Therapeutic Approach for Neuropathic Pain Relief

**DOI:** 10.3390/biomedicines11051436

**Published:** 2023-05-12

**Authors:** HyeYeong Lee, GiYoong Tae, SaeYeon Hwang, SungWon Wee, Yoon Ha, Hye-Lan Lee, DongAh Shin

**Affiliations:** 1 Spine & Spinal Cord Institute, Department of Neurosurgery, College of Medicine, Yonsei University, Seoul 03722, Republic of Korea; kimura416@yuhs.ac (H.L.); sayoon@yuhs.ac (S.H.); stephwee95@yuhs.ac (S.W.); hayoon@yuhs.ac (Y.H.); 2 School of Materials Science and Engineering, Gwangju Institute of Science and Technology (GIST), Gwangju 61005, Republic of Korea; gytae@gist.ac.kr; 3 Graduate Program in Bioindustrial Engineering, Yonsei University, Seoul 03722, Republic of Korea

**Keywords:** hydrogel, micropatch, adipose-derived stem cell, neuroregeneration, neuropathic pain

## Abstract

This study explores the therapeutic efficacy of heparin-based hydrogel micropatches containing human adipose-derived stem cells (hASCs) in treating neuropathic pain caused by nerve damage. Our results showed that hASCs exhibited neuroregenerative and pain-relieving effects when used with heparin-based hydrogel micropatches in the neuropathic pain animal model. The use of this combination also produced enhanced cell viability and nerve regeneration. We conducted various neurological behavioral tests, dynamic plantar tests, histological examinations, and neuroelectrophysiological examinations to confirm the therapeutic effect. Our findings suggest that this approach could maximize therapeutic efficacy and improve the quality of life for patients suffering from neuropathic pain.

## 1. Introduction

The International Association for the Study of Pain has defined neuropathic pain as “pain caused by a lesion or disease of the somatosensory nervous system” [1]. Neuropathic pain has been classified as an incurable disease that is associated with insomnia, depression, physical impairment, anxiety, and suicide [2,3,4]. Consequently, numerous therapeutic methods have been explored, including stem cell therapy, pharmacotherapy, neurosurgical lesioning, and anesthetic blocks. While anticonvulsants, opioids, topical agents, and antidepressants have been used in pharmacotherapy, these methods may not be suitable for long-term therapy, and anesthetic blocks also have limitations. Neurological lesioning is not an optimal treatment due to its unpredictable outcomes and high risk [5].

Therefore, stem cell therapy has emerged as a potential therapeutic method, and various stem cell types have been used to treat neuropathic pain, including embryonic, adult, and induced pluripotent stem cells (iPSCs). However, ethical concerns regarding embryonic stem cells and the tumorigenicity of iPSCs have limited their potential use in treatment [2,3]. This study focuses on adipose-derived stem cells (ASCs) because they are easily obtainable and can be obtained with minimal harm to donors. Additionally, studies have shown that human ASCs (hASCs) secrete several cytokines and neurotrophins that enhance cell survival and protect against cell degeneration [6,7]. While some adult stem cells have limitations in differentiating into specific cell lines, hASCs have been shown to differentiate into neurons, as indicated in previous studies [6].

Despite the various advantages of adult stem cell therapy, such as fewer ethical concerns and reduced tumorigenic effects, there are limitations in terms of cell survival and efficacy of cell delivery. In our previous report, we demonstrated the therapeutic effects of hASCs by epineural transplantation (ENT). From the results, we confirm that direct cell injection could promote secondary damage so ENT is a better method than injecting in sciatic nerve injury. However, the ENT has lower cell survival so it is required to use alternate method for cell delivery that could enhance cell survival as well as better outcomes [2]. Several methods have been applied to enhance the viability and engraftment of adult stem cells, including the use of supporting materials that can improve the efficiency of hASCs. For instance, a scaffold containing stem cells was used as a nerve conduit, with the stem cells cultured in vitro and assembled onto the scaffold, which was made by electrospinning or freeze drying to provide a porous structure for cell adhesion and distribution [8,9,10]. Another method involved the application of microbeads, where the cultured cells were encapsulated in micro-size beads and differentiated in vitro to the intended lineage [11]. A bulking agent composed of silk fibroin microbeads and hASCs was also developed. In our study, we utilized a heparin-based hydrogel as a carrier to enhance cell survival and delivery efficiency in injured nerve tissue. Previously, we developed a bioactive heparin-based hydrogel that functioned well as a scaffold for delivering growth factors and culturing hepatocytes and chondrocytes [12,13,14]. This heparin-based hydrogel was found to be optimal for adhesion, proliferation, maintenance of stemness, and directed differentiation, which was mediated by the affinity of hASCs for heparin [15]. A method was also proposed for preparing micropatterned heparin-based hydrogels and safely retrieving them via photo-polymerization of the hydrogels via lithography on a polyelectrolyte multilayer (PEM) with electrochemical desorption of the PEM [16]. In this study, we developed the neuropathic pain animal model by sciatic nerve crush, and used micropatches of heparin-based hydrogel as a delivery carrier for hASCs to enhance the neuropathic pain and to recover damaged sciatic nerves in rats. The functional recovery was analyzed by behavior tests, and the establishment of transplanted hASCs on micropatches at the injury site and recovery of the injured nerve were also characterized by histological analysis.

## 2. Materials and Methods

### 2.1. Materials

ITO-coated glass (1.25 cm × 1.25 cm, sheet resistance = Ω/sq, thickness = 1800 Å, transmittance ≥ 82%) was purchased from Delta Technologies (Stillwater, MN, USA). Corning^®^ Costar^®^ Ultra-Low attachment multiwell plates were purchased from Corning^®^ (Tewksbury, MA, USA). Heparin sodium salt (12 kDa, from porcine intestinal mucosa) was purchased from Cellsus Ins. (Cincinnati, OH, USA). Poly(L-lysine) (PLL, 15 kDa), acridine orange (AO), propidium iodide (PI), and glycidyl methacrylate (GMA) were obtained from Sigma-Aldrich (St. Louis, MO, USA). Hyaluronic acid (HA, 132 kDa) was obtained from Lifecore Biomedical (Chaska, MN, USA). Chitosan (Chi, water-soluble chitooligosaccharide, 5 kDa, degree of deacetylation = 85%) was purchased from Kittolife Co., Ltd. (Seoul, Republic of Korea). Poly(ethylene glycol) diacrylate (PEG-DA, 6 kDa, degree of substitution = 98%) and poly(ethylene glycol) sulfhydryl (PEG-SH, 10 kDa) were obtained from SunBio Inc. (Anyang, Republic of Korea). Dulbecco’s modified eagle medium (DMEM), fetal bovine serum (FBS), and penicillin/streptomycin were purchased from GIBCO (Grand Island, NY, USA).

### 2.2. Preparation of Polyelectrolyte Multilayer (PEM) on ITO Electrodes by Layer-by-Layer Method

(PLL-HA)2-(GMA-Chi) on ITO electrode was prepared as previously reported by us [16]. Briefly, after 10 min ultrasonication of ITO electrodes in isopropyl alcohol and deionized water for 10 min, respectively, ITO electrodes were treated by oxygen plasma (YES-R3, San Jose, CA, USA) at 300 W for 5 min. Then, they were immersed in electrolyte solutions (0.5 mg/mL PLL and HA in PBS) sequentially for 5 min with a PBS washing step between each step. After forming a bilayer of PLL-HA, glycidyl methacrylated chitosan (GMA-chitosan), produced as previously reported 35 (30% acrylation, 20 mg/mL), was adsorbed on top of it.

### 2.3. Formation of Micropatterned Heparin-Based Hydrogels on the PEM-Coated ITO Electrodes

Micropatterned heparin-based hydrogels were prepared as previously reported by us [16]. In brief, thiolated heparin (Hep-SH) and PEG-DA with the molar ratio between the thiol group in Hep-SH and acrylate group in PEG-DA of 1:0.3 were dissolved at 5.3 wt% in PBS containing 0.1 wt% Irgacure 2959 (in 70% ethanol). After pouring the precursor solution (5 μL) on PEM-modified ITO, a coverslip was placed. Then, UV light was irradiated for 10 s (18 W cm^2^, 365 nm) with a photo mask to induce the micropatterned hydrogel on the electrode. Finally, the electrode was rinsed with PBS.

### 2.4. Cell Culture and Seeding

We used hASCs (R7788115; Thermo Fisher Scientific, Waltham, MA, USA) for cell-loaded micropatches. hASCs were cultured in a 10 cm^2^ culture dish containing Alpha modified Eagle’s medium (Gibco, Grand Island, NY, USA), 10% fetal bovine serum (FBS; Gibco), and 1% penicillin–streptomycin (Gibco) at 37 °C in a humidified atmosphere of 5% CO_2_ as manufactures protocol. When cells reached 70–80% confluence, they were trypsinized with TrypLE™ Express without phenol red (Gibco), and the seeding density was 5000 cells/cm^2^. The medium was replaced every 3–4 days with fresh subculture. Cells were used for transplantation at passages 5–6.

Before seeding hASCs on the micropatterned heparin-based hydrogel, PEG-SH in PBS (pH 8.0) was grafted on the remaining area of the electrode without hydrogel pattern to prevent the non-selective cell attachment on the substrate. Then, hASCs (2 × 105 cells) were seeded on the electrode with hydrogel micropattern in a 6-well plate (ultra-low attachment). The seeded cells on the micropatterned hydrogels were cultured for 7 days in DMEM containing 10% FBS and 1% penicillin/streptomycin.

### 2.5. Retrieval of Cell-Laden Micropatches by Electrochemical Stimulation

The electrochemical retrieval of the micropatches was achieved by an electrochemical stimulation (2 V for 5 min) to the ITO by using a potentiostat (CH Instruments 660D, Austin, TX, USA). The detached micropatches were collected and centrifuged (1500 rpm, 5 min) to remove DMEM and free PEMs. The viability of the retrieved cells on the micropatches was characterized by live/dead staining using AO (0.67 μM) and PI (0.75 μM) [17].

### 2.6. Live/Dead Staining

After 3 days of cell culture, the cells were stained with a live/thread assay kit (Gibco Invitrogen, Logan, UT, USA) using Calcein AM and ethidium homodimer double staining under a fluorescence microscope (Olympus IX71) to examine hASCs attachment and viability. Calcein AM stains the cytoplasm of viable cells green, and ethidium homodimer stains the nucleus of non-viable cells red.

### 2.7. Neuropathic Pain Model and Implant of Micropatches

Based on the studies showing that combining male and female data yields poor results [18], we used male Sprague Dawley rats (200 ± 20 g, mean ± standard deviation; OrientBio, Kyungki-do, Republic of Korea) with 10–15 rats in 3 groups. The rats were housed in an animal facility permitted by the Association for Assessment and Accreditation of Laboratory Animal Care (AAALAC). For inducing sciatic nerve crush injury, all animals were anesthetized with Zoletil 50 (25 mg kg^−1^, Virbac Laboratories, Carros, France) and Rompun (10 mg kg^−1^, Bayer Korea, Seoul, Republic of Korea). The muscle of the left leg of the anesthetized rat was incised, and damage to the sciatic nerve was induced using a 100 g vessel clip at 1 cm proximal from the division site of tibial and peroneal nerve (18055-04; Fine Science Tool, Foster City, CA, USA) for 10 min. After 1 week of sciatic nerve crush, the injured sciatic nerve was exposed and PBS solution (100 μL), hASCs (1 × 10^6^ cells/100 μL PBS), or hASCs + hydrogel in 100 μL PBS was applied on the epineurium of the injured sciatic nerve (ENT) [2]. Animals were blindly grouped by different investigator. After surgery, cefazolin (25 mg/kg, Chong Kun Dang, Seoul, Republic of Korea) was injected daily for 5 days and cyclosporine (20 mg/kg, Chong Kun Dang, Seoul, Republic of Korea) was injected until sacrificed. All experimental procedures were performed according to protocols approved by the directives of the Institutional Animal Care and Use Committee (IACUC; Protocol number: 2012-0271).

### 2.8. Sciatic Functional Index Analysis

For the analysis of SFI, the hind paws of the animals were painted with non-toxic finger paint (Dong-a Pencil Co., Ltd., Daejeon, Republic of Korea), and animals walked through a 70 cm × 19 cm × 12 cm walking track with white paper. The length of three points was measured from the footprints of the left leg of sham (N) and the left leg of the experimental group (E); the measurement values from the 1st to 5th toe, toe spread (TS); the measurement values of paw length (PL); the measurement values from the 2nd to 4th toe, intermediate toe spread (IT). Toe SFI was calculated using the Bain et al. formula [19,20], and SFI values were measured weekly.

### 2.9. Immunohistochemistry

At 5 weeks after injury, the animals were sacrificed with CO_2_ and intracardially perfused with 4% paraformaldehyde (in PBS). For immunohistochemistry, left leg sciatic nerves and lumbar 4–5 level left side dorsal root ganglion (DRG) were excised for immunohistochemistry, fixed in 4% paraformaldehyde (in PBS) for 24 h, and stored in 30% sucrose (in PBS). After embedding and freezing with optimal cutting temperature compound (Sakura Finetek, Torrance, CA, USA), samples were sliced into 20 μm thick coronal sections centered on the injured area.

The sectioned samples were attached to slides, washed with PBS, and blocked with 10% normal donkey serum at room temperature for 1 h. After blocking, samples were treated with mouse anti-Human nuclei (1:100, Millipore, Billerica, MA, USA) for hASCs detection and rabbit anti-βIII tubulin (1:1000, abcam, Cambridge, MA, USA) for neuronal cell, chicken anti-vimentin (1:4000, Millipore) for Schwann cell, mouse anti-Calcitonin Gene-Related Peptide (CGRP; 1:100, abcam) for pain-related marker, and chicken anti-neurofilament medium (NF; 1:2000, abcam) for mature neuron incubated overnight at 4 °C with a diluted primary antibody. After washing 3 times by PBS, samples were incubated with CyTM3-donkey anti-chicken IgG (1:500, Bar Harbor, ME, USA), CyTM3-donkey anti-rabbit IgG (1:500 Jackson), and FITC-donkey anti-mouse IgG (1:200, Jackson) at room temperature for 1 h). For nuclear staining, a 4′,6-diamidino-2-phenylindole (DAPI)-conjugated mounting medium (Vector Laboratories, Peterborough, UK) was used. Samples were observed by confocal microscopy (LSM 700, Carl Zeiss, Oberkochen, Germany). All types of histological analysis were performed 5 weeks after injury when behavioral assessments reached certain criteria in the Ctrl group and our previous study as a reference [2]. The number of samples used for IHC was plotted as a scatter plot.

### 2.10. Transmission Electron Microscopy (TEM) and Toluidine Blue Staining

At 5 weeks after injury, the sciatic nerve was isolated and pre-fixed by Karnovsky’s solution (2% PFA, 2% glutaraldehyde, and 0.1 M phosphate buffer, Millipore) for 6 h. After washing, samples were post-fixed by 1% osmium tetroxide (OsO4, Polysciences, Warrington, PA, USA) in 0.1 M phosphate buffer for 2 h. Fixed samples were dehydrated in ascending gradual series (0%, 60%, 70%, 80%, 90%, 95%, and 100%) and infiltrated by propylene oxide. Infiltrated samples were embedded in a mixture of propylene oxide and Poly/Bed 812 kit (Polysciences) overnight in an electron microscope oven (TD-700, DOSAKA, Kyoto, Japan). Samples were cut into 250~300 nm thickness sections by an ultra-microtome (Leica ultracut UCT, Vienna, Austria). The sectioned samples were stained with 1% toluidine blue (Sigma-Aldrich, St. Louis, MO, USA) and cut into 80~100 nm thickness sections by a diamond knife (Diatome, Biel, Switzerland). The thin-sectioned samples were double stained by 6% uranyl acetate (EMS 22400, Hatfield, PA, USA, for 10 min) and lead citrate (Fisher Scientific, Vantaa, Finland, for 10 min) for contrast staining on a copper grid and examined by TEM (JEM-1011/Megaview III, JEOL, Tokyo, Japan).

### 2.11. Mechanical Allodynia Measurement

For measurements of mechanical allodynia, rats were stabilized in an acrylic box with a wire mesh platform for 15 min. Using a dynamic plantar aesthesiometer (Ugo Basile, Comerio, Varese, Italy), the plantar paw was stimulated with a 0.5 mm diameter steel rod with gradually increasing force from 0 to 50 g for 20 s. When the animal withdrew the hind paw, the stimulation of the horizontal bar was automatically stopped, and the force was recorded. Hind paw values were measured weekly 4 or more times for each hind paw with an interval of 5 min or longer between each stimulation.

### 2.12. Electrophysiological Study

Motor-evoked potentials (MEPs) (*n* = 15 each) and somatosensory-evoked potentials (SSEPs) (*n* = 15 each) were measured at 5 weeks after injury. Animals were anesthetized, and stimulating electrodes were inserted into the parietal and masticatory muscle for MEPs measurement, and recording electrodes were fixed to the left leg biceps femoris muscles [21]. For the SSEPs measurement, a 4 × 4 mm size craniectomy was performed, and the recording electrode (NE-120, Rhodes Medical Instruments, Tujunga, CA, USA) was slightly stuck in the sensorimotor cortex at 2 mm posterior and 2 mm lateral to the bregma. For stimulation, the left sciatic nerve was exposed and fixed by a bipolar stimulating electrode (730338, Harvard apparatus, Holliston, MA, USA). MEP was measured at 0 mA, 6 mA, 9 mA, 15 mA, 18 mA, and 20 mA (Figure S1B). SSEP was measured at 0 mA, 0.01 mA, 0.1 mA, 0.5 mA, 1 mA, and 3 mA (Figure S1A). From 20 mA of MEP and 3 mA of SSEP records, we obtained latency to initial response, positive and negative peak, and amplitude of each peak. Stimulation was produced continuously and constantly by a train/delay generator (DG2A, Digimeter Ltd., Welwyn Garden City, UK) and transferred to animals through the current stimulator (DS3, Digimeter Ltd.). The transferred stimulation generated evoked potentials. A bioamplifier (Axoclamp 900A, Molecular Devices, Union City, CA, USA) amplified the signal, and it was analyzed by software (qCLAMP 10, Molecular devices).

### 2.13. Statistical Analysis

All data are expressed as mean ± standard deviation. One-way analysis of variance with multiple comparisons and Tukey assay (*, *p* < 0.05; **, *p* < 0.01; and ***, *p* < 0.001) was performed. A *p*-value of <0.05 was considered statistically significant. Graphs were created, and statistical analysis was performed using GraphPad PRISM 9.5.1 (GraphPad Software Inc., San Diego, CA, USA).

## 3. Results

### 3.1. Heparin-Based Hydrogel Micropatches Enhance the Viability of Adherent hASCs

**Figure 1:**
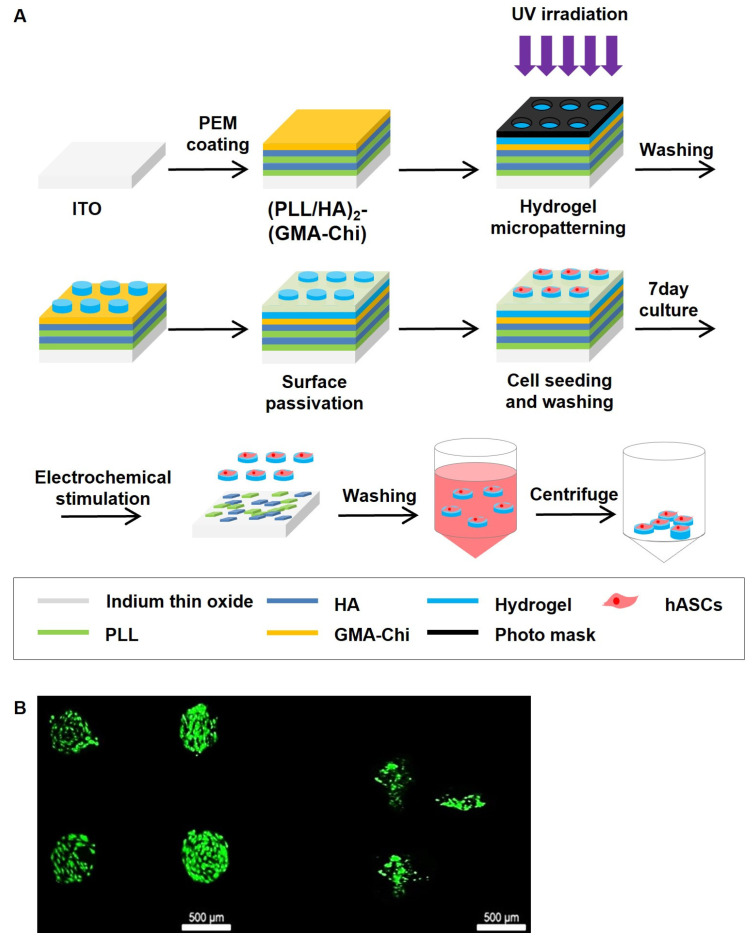
Effects of hASCs + micropatches production process and the whole process on cell survival. (**A**) Scheme of the preparation of hASCs-attached micropatterned heparin-based hydrogel by photo-polymerization on polyelectrolyte multilayer (PEM) and the retrieval of hASCs-attached micropatches by electrochemical stimulus. (**B**) Live/dead staining of hASCs on heparin-based hydrogel. The left figure is hASCs on micropatterned hydrogels after 7 days of seeding. The right figure is hASCs-attached micropatches retrieved by electrochemical stimulus. The whole process does not significantly affect hASCs survival.

To improve the therapeutic effect, survival, and successful engraftment of transplanted hASCs in the injured nerve, we employed heparin-based hydrogel micropatches. Micropatches containing heparin-based hydrogel and hASCs were produced using a previously reported method [16]. First, a PEM composed of poly(L-lysine)-hyaluronic acid-2-glycidyl methacrylated chitosan was formed on an electrode using a layer-by-layer method. Then, the micropatterned heparin-based hydrogel was formed by photo-polymerization of thiolated heparin and poly(ethylene glycol) diacrylate (PEG) on top of the PEM via lithography. The remaining area was passivated by PEG grafting using thiolated PEG. Next, hASCs were seeded and cultured for 7 days. Then, hASCs adhered to micropatches of the heparin-based hydrogel were obtained using electrochemical stimulation (2 V for 5 min) (Figure 1A).

To characterize the biocompatibility of the present method of obtaining hASC-containing micropatches, the cell viability of the seeded hASCs was analyzed using live/dead staining. As expected, seeded, living hASCs were selectively located on the micropatterned heparin-based hydrogels because of their affinity for heparin [17,22] (Figure 1B), indicating that the micropatterned heparin-based hydrogel is biocompatible and PEG grafting (surface passivation) successfully prevented the non-specific attachment of seeded hASCs. More importantly, after retrieving the cell-laden micropatches using an electrochemical stimulus, the micropatches were not aggregated with each other, and the hASCs were still alive on size-controlled micropatches (Figure 1B), confirming the absence of cytotoxic effects of the electrochemical retrieval method on hASCs.

Thus, the overall process of obtaining hASC-attached micropatches was compatible with in vivo application, and several hundred-micrometer micropatches containing stabilized hASCs were obtained for further experiments.

### 3.2. Implanted hASC Micropatches Restore Injured Sciatic Nerves

**Figure 2:**
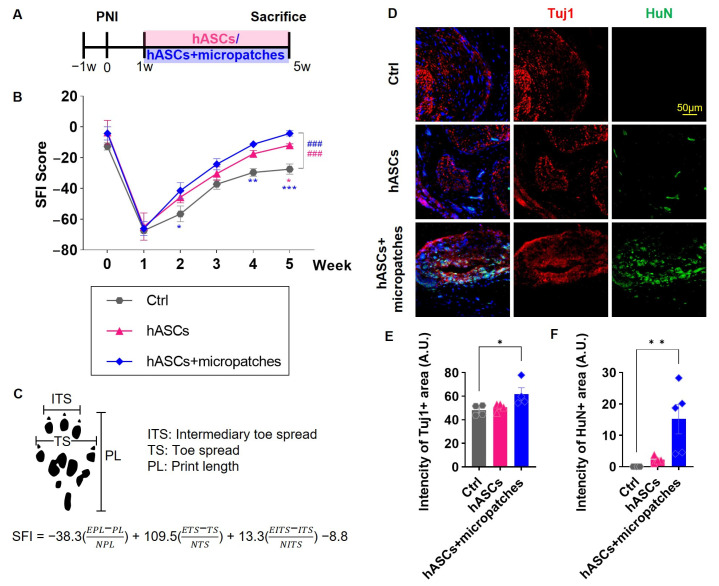
Effect of hASC + micropatches on recovery and regeneration of neuronal function. (**A**) Timeline observed from 1 week after sciatic nerve injury surgery up to 4 weeks post-treatment with hASCs and hASC + micropatches. (**B**) Sciatic function index score graph by the group. Due to nerve injury, SFI scores were reduced, and hASC transplantation restored some functions. This enhanced therapeutic efficacy when implanted in the form of hASCs + micropatches. (**C**) Criteria and formulas for measuring SFI scores on soles. (**D**) Immunohistochemistry of the sciatic nerve in each group with anti-Tuj1 (red) and HuN (green) antibodies and DAPI at post injury (PI) 5 weeks. (**E**) Mean intensity of the Tuj1 in each group. (**F**) Mean intensity of the HuN in each group. hASCs + micropatches the recovery of Tuj1, which was decreased after injury. Especially, hASCs+ micropatches significantly differed from Ctrl by increasing hASCs (+HuN) survival. All data are expressed as mean ± S.E. Multiple comparisons results expressed by * *p* < 0.05; ** *p* < 0.01; *** *p* < 0.001, and analysis by linear mixed effects model is expressed to ### *p* < 0.001.

The neuropathic pain models induced a marked impairment of nerve function, as evidenced by abnormal findings such as shrinking of the spread sole and walking with the whole foot instead of on the toes. However, transplantation of hASCs or hASCs + micropatches one week after injury was able to restore neuronal function. Of note, the hASCs + micropatches group demonstrated significantly better recovery than the control group, with maximal recovery potential observed during the final week of observation (Figure 2A–C). Although hASCs+ micropatches were not significantly different from hASCs, there was a significant difference between the Ctrl group and statistical significance, which was very encouraging.

The histological findings showed the same results. Injured nerves exhibited greatly reduced Tuj1 expression, and transplantation of hASCs or hASCs + micropatches restored Tuj1 expression levels (Figure 2D,E). This finding can be explained in relation to the survival of transplanted cells. Although some transplanted cells were observed in the group transplanted with hASCs alone, many HuN-positive hASCs were observed in the group transplanted with hASCs and micropatches (Figure 2D,F). Taken together, these results suggest that hASCs can induce the repair of damaged nerves and enhance their survival when transplanted in combination with micropatches, ultimately accelerating functional recovery.

### 3.3. Transplanted hASC Micropatches Activate Remyelination in Injured Sciatic Nerves

**Figure 3:**
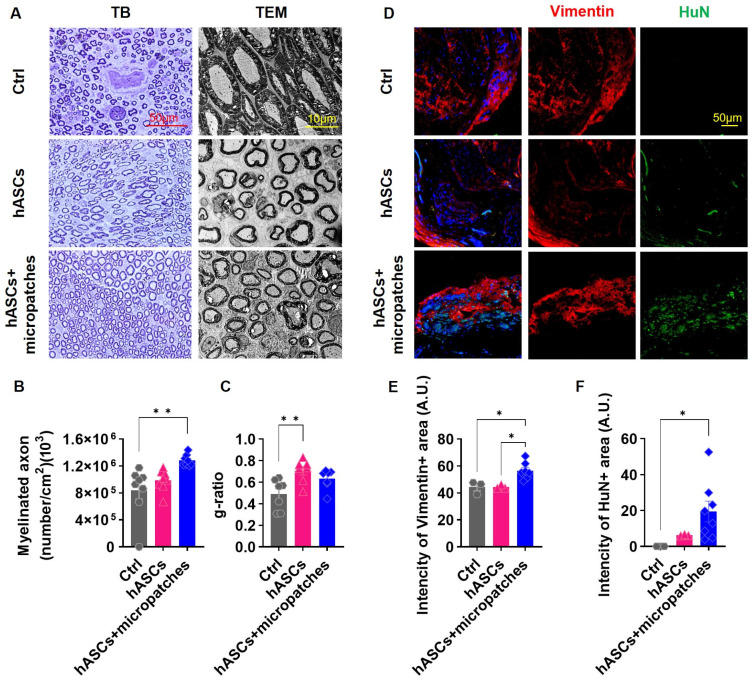
Implanted hASCs micropatches activate the remyelination of injured sciatic nerves. (**A**) Toluidine blue (TB) staining and Transmission electron microscopy (TEM) images of the injury site of the sciatic nerve in each group at PI 5 weeks. (**B**) Density of myelinated axons in each group at PI 5 weeks in TB staining of the sciatic nerve. Transplanted hASCs + micropatches ameliorate the number of myelinated axons that were reduced by injury. (**C**) Calculated g-ratio in each group at PI 5 weeks in TEM images. The transplanted hASCs micropatch restored the nerve to the g-ratio, where the sciatic nerve functions normally. (**D**) Immunohistochemistry of the sciatic nerve in each group with anti-vimentin (red) and HuN (green) antibodies and DAPI at post injury (PI) 5 weeks. (**E**) Mean intensity of the vimentin in each group. (**F**) Mean intensity of the HuN in each group. hASCs + micropatches increased Schwann cells (+vimentin) by increasing hASCs (+HuN) survival. All data are expressed as mean ± S.E. * *p* < 0.05; ** *p* < 0.01.

Nerve injury involves damage to the myelin that enwraps axons. Demyelinated damaged nerves aggravate neuropathic pain. Aberrant morphologies of myelinated axons were observed in injured nerve tissue along with extended axon-to-axon spacing. Transplantation of hASCs or hASCs + micropatches restored the normal morphology and induced the production of new myelinated axons. The greatest increase in the number of myelinated axons was observed when hASCs + micropatches were transplanted (Figure 3A,B).

For myelinated axons to have ideal velocity and conductance, the axons should be wrapped at an appropriate thickness. This measure can be expressed as the g-ratio, which is the axonal diameter divided by the fiber diameter, and a suitable g-ratio for the sciatic nerve is in the range of 0.55–0.68 [23]. Measurement of the g-ratio by transmission electron microscopy images showed a low value because the axons were wrapped excessively due to nerve damage. Transplantation of hASCs normalized some of the aberrant morphologies and thickly wrapped axons. However, rather than an appropriate thickness, thin wrapping, and large g-ratios were observed. Transplantation of hASCs and micropatches overcame this problem and restored the adequate thickness of myelinated axons (Figure 3A,C).

Similar results were observed in immunohistochemical analysis. Transplanted hASCs were most frequently observed when hASCs + micropatches were transplanted, and vimentin, which was used as a Schwann cell marker, was also most highly observed in this group (Figure 3D–F). Taken together, these results demonstrate that transplantation of hASCs + micropatches, but not hASCs alone, activates myelination, which is important for alleviating neuropathic pain to allow for proper neuronal function.

### 3.4. Implanted hASC Micropatches Reduce Neuropathic Pain

**Figure 4:**
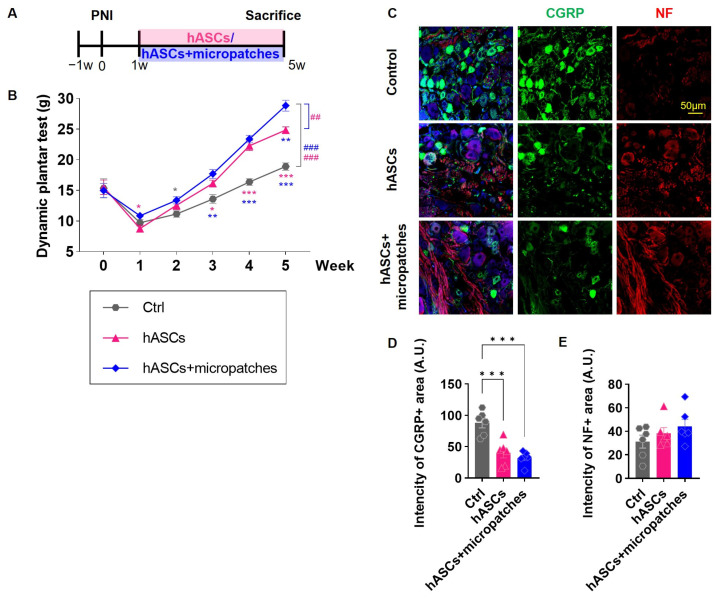
Transplanted hASCs micropatches relieve neuropathic pain and reduce the expression of related factors. (**A**) Timeline observed from 1 week after sciatic nerve injury surgery up to 4 weeks post-treatment with hASCs and hASC + micropatches. (**B**) Dynamic plantar test of each group for a total of 5 weeks. Mechanical allodynia was statistically significantly restored in the Ctrl group after transplantation of hASCs and hASCs + micropatches. (**C**) Immunohistochemistry of the dorsal root ganglion in each group with anti-CGRP (green) and NF (red) antibodies and DAPI at post injury (PI) 5 weeks. (**D**) Mean intensity of the CGRP in each group. (**E**) Mean intensity of the NF in each group. hASCs + micropatch decreased the expression of CGRP, a pain-related factor, and increased the expression of NF, a neuronal marker. All data are expressed as mean ± S.E. Multiple comparisons results expressed by * *p* < 0.05; ** *p* < 0.01; *** *p* < 0.001, and analysis by linear mixed effects model is expressed to ## *p* < 0.01; ### *p* < 0.001.

Sciatic nerve injury causes neuropathic pain. Mechanical allodynia, in which even a small force applied to the sole of the injured foot is perceived as pain, was observed. Transplantation of hASCs relieved this pain. Starting at 2 weeks after transplantation, pain was alleviated compared with that in the Ctrl group, and this effect was more pronounced when both hASCs and micropatches were transplanted (Figure 4A,B).

Neuropathic pain is associated with increased expression of calcitonin gene-related peptide (CGRP), a pain-related factor that is associated with loss of dorsal root ganglia (DRG) neurons. Nerve injury decreased NF expression in DRG tissue and increased neuropathic pain, and transplantation of hASCs, especially hASCs and micropatches, overcame this effect (Figure 4C,E). Furthermore, CGRP expression, which increased with neuropathic pain, was sharply reduced by transplantation of hASCs and hASCs + micropatches (Figure 4C,D). These results indicated that transplantation of hASCs, especially hASCs + micropatches, could alleviate neuropathic pain.

### 3.5. Implanted hASCs Micropatches Restore Neural Activity

Motor-evoked potentials (MEPs) indicate the electrical activity of muscles activated by nerve impulses and can identify and determine the extent of nerve damage affecting muscle function. Somatosensory-evoked potentials (SSEPs) indicate electrical activity produced by sensory pathways in response to stimuli and can identify nerve damage that affects sensory function, such as numbness or tingling. Similar to MEPs, the degree of nerve damage can be confirmed using SSEPs. Neuropathic pain is a condition in which nerve damage or dysfunction causes chronic pain, and MEPs and SSEPs can be used to identify the location and extent of nerve damage in these patients. In this study, we compared and analyzed the recovery of electrical nerve function by stimulating the same nerve site using the same magnitude of stimulation. The MEP and SSEP amplitudes were measured from each initial deflection to the baseline, and peak-to-peak amplitudes were measured from negative to positive deflections.

**Figure 5:**
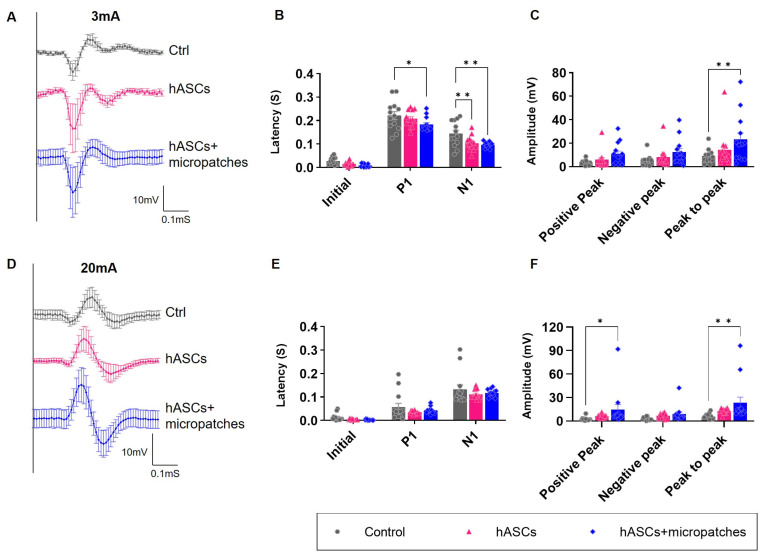
Implanted hASCs micropatches restore electrical neuronal activity. (**A**) SSEP signals by the group for 3 mV input 4 weeks after transplantation. (**B**) Latency measurement graph in SSEP signals. The initial latency of the nerve response after input and the time of positive peak and negative peak is delayed due to nerve damage. Response time was shortened when hASCs were transplanted in the form of micropatches. (**C**) Amplitude measurement graph in SSEP signals. The amplitude of sensory nerve response was also observed to be greatest with hASCs + micropatches transplantation. (**D**) MEP signals by the group for 20 mV input 4 weeks after transplantation. (**E**) Latency measurement graph in MEP signals. Delayed latency values due to nerve injury resulted in shortened response times when implanted in the form of hASCs + micropatches. (**F**) Amplitude measurement graph in MEP signals. The amplitude of motor nerve response was also observed to be greatest with hASCs + micropatches transplantation. All data are expressed as mean ± S.E. * *p* < 0.05; ** *p* < 0.01.

Transplantation of hASCs reduced the latency of SSEPs. In particular, transplantation of hASCs + micropatches caused nerves to respond very quickly to electrical stimulation (Figure 5A,B). Responses to electrical stimulation were greatly diminished upon nerve injury. Transplantation of hASCs increased the response, and a greater effect was observed when hASCs + micropatches were transplanted. The same effect was observed for both SSEPs and MEPs (Figure 5A,C,D,F). These results suggest that nerve injury reduces the response of nerves to electrical stimulation, but hASCs + micropatches can effectively restore not only the response latency to the stimulation but also the amplitude when transplanted to the injured site.

## 4. Discussion

Peripheral nerve injury is associated with neuropathic pain and abnormal nerve function at the injury site. In this study, we presented a method to effectively deliver hASCs to repair damaged nerve tissue and reduce pain. Previous studies confirmed the therapeutic effect of hASCs on neuropathic pain caused by nerve damage [2,24,25,26].

However, despite these therapeutic effects, the survival of transplanted cells is difficult to achieve. The viability of transplanted cells is critical for tissue regeneration and pain treatment because it affects the survival, proliferation, and differentiation of the transplanted cells. A study examining the effect of transplanted neural stem cells (NSCs) on neuropathic pain relief in rats reported that high survival rates of transplanted NSCs were associated with improved pain relief outcomes.

From this viewpoint, it may be advantageous to use hASCs as therapeutic agents. hASCs can be isolated from liposuction and surgical waste, which are less invasive procedures, and large numbers of cells can be produced without ethical issues or serious harm to the donor [27]. hASCs also suppress the immune system through the secretion of cytokines and chemokines, which can prevent the rejection of transplanted cells by the recipient’s immune system [28,29]. This attribute makes hASCs a promising option for allogeneic cell transplantation in which the donor and recipient are not genetically identical. In addition, hASCs may be suitable candidates for cell therapeutic agents because they promote angiogenesis and wound healing, have regenerative effects, and can differentiate into various cell types, including adipocytes, osteocytes, and chondrocytes [30,31,32,33,34,35]. 

We used hydrogel-based micropatches to take advantage of hASCs as cell therapy agents. Hydrogels have high water content and can mimic the environment of living tissue, making them a commonly used material for biomedical applications. Hydrogels can serve as scaffolds or matrices that support stem cell growth and proliferation [36]. This helps stem cells grow, differentiate into desired cell types, and regenerate damaged tissue. Hydrogel matrices can also protect stem cells from harsh environmental conditions, such as low oxygen levels, and improve cell viability. In our previous reports, we demonstrated that heparin-based hydrogel enhances cell proliferation and migration. In addition, F-actin is known to be essential for axon extension, has generated [17]. Based on the findings, we were able to hypothesize that some transplanted hASCs could directly differentiate into neuronal lineage cells, and the heparin-based hydrogel could affect in migration of other original cells. In addition, it is known that hASCs can release several types of neurotrophic factors (NTF), including NGF, BDNF, NT-3, NT-4, GDNF, and CNTF [37,38,39]. These NTFs can enhance axonal regeneration and outgrowth. Therefore, hASCs, seeded on heparin-based hydrogel, have shown a synergetic effect for the enhancement of neuropathic pain. In our results, we have shown the remyelination effect on the hASCs transplanted group along with the optimal myelin g-ratio compared to the Ctrl group. In addition, as mentioned above, hASCs have the potential to release several NTFs, and the released GDNF and BDNF could possibly induce myelination [40,41,42]. 

There has been a study that the OCT4 expression of hASCs seeded on heparin-based hydrogels has increased, and it is also known to induce re-myelination. [17,43]. Hydrogels can be designed to release growth factors and other bioactive molecules that promote stem cell differentiation, migration, and tissue regeneration. A recent study showed that stem cell-loaded hydrogel scaffolds were able to promote spinal cord tissue regeneration in rats with spinal cord injury [44,45]. Another study showed that hydrogels containing stem cells could improve cardiac function in rats with cardiac injury [46].

The use of hydrogels designed to aid in cell therapy can maximize therapeutic efficacy. The heparin-based hydrogel micropatches that we used helped to improve cell retention by creating a microenvironment that promotes attachment and proliferation of co-implanted cells. This effect can improve overall cell survival and the efficiency of the cell transplantation process. Additionally, heparin is a molecule that occurs naturally in the body and is biocompatible. As a result, heparin-based hydrogel micropatches are relatively biocompatible and can support the attachment and proliferation of various cell types, including stem cells. Heparin-based hydrogel micropatches have a high loading capacity for cells and therapeutic agents; therefore, even small amounts can deliver significant numbers of cells or therapeutic agents. This effect makes these micropatches an efficient option for cell transplantation because the total number of cells required for transplantation can be reduced. Additionally, the micropatches are easy to handle and can be applied locally to the site of cell transplantation, making cell therapeutics more efficient.

In current EP techniques, time domain parameters (latency and amplitude) are used to determine the injury. Amplitude waveforms reflect intact axons within neural pathways. Decreased amplitude can lead to axon damage or functional loss [47]. Previous experimental studies have proved that under conditions of neurological injury, EP response diminishes, causing a decrease in amplitude and an increase in latency [48]. If hypoxia progresses to a level that can cause ischemia to neurological tissues, it decreases SSEP amplitude and increases latency. In our previous study, we proved hASCs’ therapeutic effect by epidural soaking and that heparin-based micropatch is a useful method for neuroregeneration by increasing the survival of hASCs. Treatment of hASCs with heparin-based hydrogel micropatches significantly improved neurological function, which was not significantly different from the Ctrl group.

Neuropathic pain is a common medical disorder that affects millions of people worldwide. There are many reasons, such as traffic accidents, sports accidents, infection, and degenerative neurological disorders, that cause neuropathic pain. In this study, we confirmed the therapeutic effect of treating neuropathic pain caused by nerve damage that could occur due to various accidents by using heparin-based hydrogel micropatch with hASCs. Based on our findings, it could be suggested as a future therapeutic method for neuropathic pain patients. hASCs produced neuroregeneration and pain-relieving effects, and when used with heparin-based hydrogel micropatches, they exhibited increased cell viability and maximized therapeutic efficacy. Future studies will be performed to identify the precise mechanisms by which heparin-based hydrogel micropatches affect cells.

## Data Availability

The data presented in this study are available in this article.

## Supplementary Materials

The following supporting information can be downloaded at: https://www.mdpi.com/article/10.3390/biomedicines11051436/s1, Figure S1: Records of MEP and SSEP by input. (A) SSEP signals by group from 0.01 mV input to 3 mA at 4 weeks after transplantation. (B) MEP signals by group from 6 mA input to 20 mA at 4 weeks after transplantation.
