# Supplementary material for: Heparin-Based Hydrogel Micropatches with Human Adipose-Derived Stem Cells: A Promising Therapeutic Approach for Neuropathic Pain Relief
Source: Biomedicines. 2023 May 12;11(5):1436. doi: 10.3390/biomedicines11051436 (PMC10216470; doi:10.3390/biomedicines11051436)
Supplement: Supplementary file 1 [file biomedicines-11-01436-s001.zip › biomedicines-2371199-supplementary.pdf]

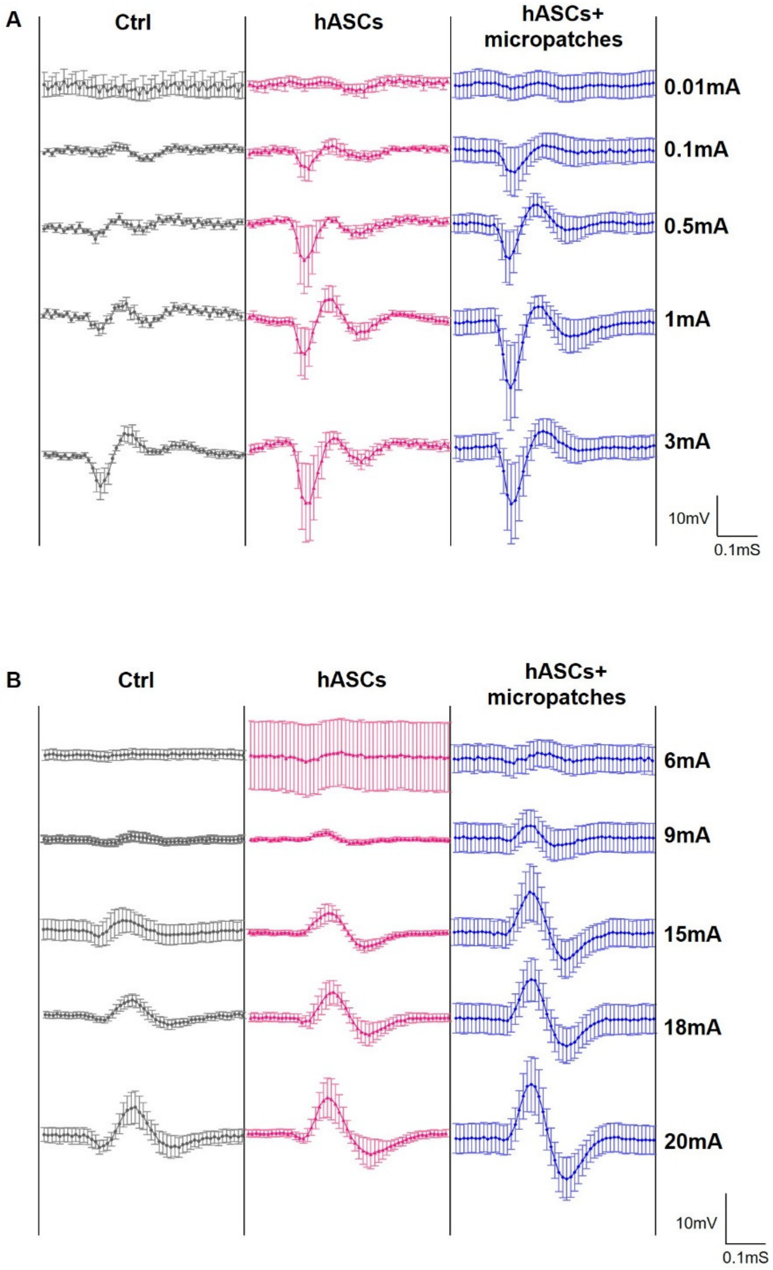

Figure S1. Records of MEP and SSEP by input. (**A**) SSEP signals by group from 0.01mV input to 3mA at 4 weeks after transplantation. (**B**) MEP signals by group from 6mA input to 20mA at 4 weeks after transplantation.
